# Skeletal Muscle Density as a Predictive Marker for Pathologic Complete Response in Triple-Negative Breast Cancer Treated with Neoadjuvant Chemoimmunotherapy

**DOI:** 10.3390/cancers17111768

**Published:** 2025-05-25

**Authors:** Han Song Mun, Sung Hun Kim, Jieun Lee, Se Jun Park, Ahwon Lee, Jun Kang, Woo-Chan Park, Soo Youn Bae, Byung Ok Choi, Ji Hyun Hong, Soon Nam Oh, Kabsoo Shin

**Affiliations:** 1 Department of Radiology, Seoul St. Mary’s Hospital, College of Medicine, The Catholic University of Korea, Seoul 06591, Republic of Korea; im_hsm@catholic.ac.kr (H.S.M.);; 2 Division of Medical Oncology, Department of Internal Medicine, Seoul St. Mary’s Hospital, College of Medicine, The Catholic University of Korea, Seoul 06591, Republic of Korea; 3 Cancer Research Institute, College of Medicine, The Catholic University of Korea, Seoul 06591, Republic of Korea; 4 Department of Hospital Pathology, Seoul St. Mary’s Hospital, College of Medicine, The Catholic University of Korea, Seoul 06591, Republic of Korea; 5 Department of Surgery, Seoul St. Mary’s Hospital, College of Medicine, The Catholic University of Korea, Seoul 06591, Republic of Korea; 6 Department of Radiation Oncology, Seoul St. Mary’s Hospital, College of Medicine, The Catholic University of Korea, Seoul 06591, Republic of Korea

**Keywords:** triple negative breast neoplasm, muscle, skeletal, neoadjuvant therapy, biomarkers, pathologic complete response

## Abstract

**Background**: The predictive value of muscle-related indicators in triple-negative breast cancer (TNBC) patients undergoing neoadjuvant chemotherapy (NAC) remains unclear. This study aimed to evaluate the association between the skeletal muscle density (SMD) and clinical variables related to the physical reserve with respect to its impact on the pathologic complete response (pCR). **Methods**: We retrospectively analyzed TNBC patients who underwent NAC at Seoul St. Mary’s Hospital, Catholic University of Korea, from March 2021 to March 2024, via receiving paclitaxel/carboplatin followed by doxorubicin/cyclophosphamide, with or without pembrolizumab. Muscle indices were assessed from CT measurements of the entire cross-sectional muscle area at the L3 level using commercial deep learning software (ClariMetabo version 1.03). **Results**: A total of 144 patients were included, where 102 received chemoimmunotherapy (NACIT) and 42 received chemotherapy alone (NACT). A higher SMD was significantly associated with a younger age, lower BMI, and fewer comorbidities. In the NACIT group, patients in the high-SMD group (n = 68) demonstrated a higher relative dose intensity (*p* = 0.003) and improved pCR rates (63.2% vs. 44.1%, *p* = 0.066) compared with the low-SMD group (n = 34). The multivariable regression analysis identified a higher SMD (per 5-unit increment: OR = 1.67, *p* = 0.003) and increased PD-L1 combined positive score (per 10-unit increment: OR = 1.38, *p* = 0.019) as independent predictors of a pCR. The event-free survival was significantly longer in the high-SMD group (*p* = 0.017) and among patients that achieved a pCR (*p* < 0.001). In the NACT group, the SMD was not associated with a pCR or survival. **Conclusions**: The CT-measured SMD reflected the physical reserve in the TNBC patients that received NAC. Alongside the CPS, SMD may serve as a predictive marker for NACIT efficacy.

## Simple Summary

In patients receiving intensive cancer treatment, both tumor-related and host-related factors influence the treatment outcomes. The physical reserve is a key host factor, but objective ways to measure it are limited. In high-risk, early-stage, triple-negative breast cancer (TNBC), neoadjuvant chemoimmunotherapy (NACIT) is the standard of care. This study examined whether the skeletal muscle density (SMD), measured by CT imaging, reflected the physical reserve and predicted the treatment response. A higher SMD was associated with a younger age, fewer comorbidities, higher rates of pathologic complete response (pCR), and better survival in the patients that received NACIT. In addition, the PD-L1 expression, as measured by the combined positive score (CPS), was an independent predictor of a pCR. Both the SMD and CPS independently predicted a pCR. These findings suggest that SMD is an objective biomarker of physical reserve and treatment efficacy. Together with the CPS, it may help guide personalized treatment strategies and highlights the importance of host-related factors in optimizing outcomes with NACIT.

## 1. Introduction

Triple-negative breast cancer (TNBC) accounts for approximately 15% of all breast cancer cases and is defined by the absence of estrogen receptors (ERs), progesterone receptors (PRs), and human epidermal growth factor receptor 2 (HER2) [1]. Clinically, TNBC is characterized by an aggressive disease course, with a higher risk of early recurrence and distant metastasis compared with other breast cancer subtypes [2]. The limited availability of targeted therapies presents significant challenges in treatment, making TNBC one of the most difficult subtypes to manage.

Given its inherently systemic nature, even in the early stages, systemic chemotherapy remains the mainstay of TNBC treatment. In stages II–III of TNBC, neoadjuvant chemotherapy (NAC) is the preferred approach for disease control with downstaging and prognostification [3,4,5]. Achieving a pathologic complete response (pCR) following NAC is considered a key marker of treatment efficacy and is strongly associated with improved long-term outcomes in TNBC [6]. Additionally, PD-L1 expression and stromal tumor-infiltrating lymphocytes (TILs), which are tumor microenvironment-related biomarkers, are recognized as indicators for predicting a pCR [7,8,9].

Beyond tumor-related factors, host factors reflecting a patient’s physical reserve, such as age, ECOG performance status (PS), and comorbidities, can influence the NAC toxicity and dose intensity. These factors may also impact the pCR and overall prognosis [10,11,12,13]. However, in high-risk TNBC, patients eligible for NAC are typically younger individuals with fewer comorbidities and an ECOG PS of 1 or lower. As a result, conventional host factors alone may be insufficient to accurately assess their physical reserve, highlighting the need for additional indicators.

Skeletal muscle, the largest organ in the human body, plays a crucial role in physical function, metabolism, and inflammation regulation. Various skeletal muscle-related indices have been investigated as host factors reflecting the comorbidities and performance status in cancer patients [14,15,16,17,18]. Among these, the skeletal muscle index (SMI), which represents the muscle quantity, and skeletal muscle density (SMD), which reflects the muscle quality, are widely studied due to their relatively simple measurement using imaging techniques [19]. The SMI and SMD have been associated not only with cancer prognosis but also with treatment response and tolerance [20,21].

Recent studies have reported their relevance in both metastatic and early-stage breast cancer, linking the SMI and SMD to prognosis and treatment-related toxicities [22,23,24,25,26]. However, evidence directly associating these skeletal muscle indices with the treatment response in the NAC setting remains insufficient. Additionally, several studies have suggested a potential association between the efficacy of PD-1 inhibitors and factors such as muscle mass, muscle quality, and obesity [27,28,29,30,31,32]. However, in breast cancer patients receiving combination therapy with chemotherapy and PD-1 inhibitors, the impact of muscle-related indices on treatment outcomes remains largely unknown.

Based on this background, we hypothesized that muscle-related factors serve as indicators of physical reserve in patients with breast cancer undergoing NAC and are associated with tolerance to intensive treatment, ultimately influencing the treatment response and survival.

Therefore, this study aimed to evaluate the impact of muscle-related factors on the pCRs and survival outcomes in two regimens from the KEYNOTE-522 (KN-522) trial: a platinum-containing regimen that comprised carboplatin and paclitaxel followed by doxorubicin and cyclophosphamide, and a more intensive regimen that incorporated the PD-1 inhibitor pembrolizumab [33].

## 2. Materials and Methods

### 2.1. Patients

We retrospectively enrolled patients with TNBC who underwent NAC at Seoul St. Mary’s Hospital, Catholic University of Korea, between March 2021 and March 2024. This study’s eligibility criteria included (1) histologically confirmed TNBC; (2) stage II–III disease; and (3) neoadjuvant treatment consisting of paclitaxel/carboplatin followed by doxorubicin/cyclophosphamide, with or without pembrolizumab.

This study was approved by the Institutional Review Board of Seoul St. Mary’s Hospital, Catholic University of Korea (KC24RISI0533).

### 2.2. Skeletal Muscle Evaluation

The skeletal muscle radiodensity was assessed using abdominopelvic CT images obtained at the time of diagnosis. The cross-sectional areas of the rectus, transverse, and oblique abdominal muscles; psoas muscles; and paraspinal muscles were measured at the L3 vertebral level using commercial deep learning-based software (ClariMetabo version 1.03; ClariPi, Seoul, Republic of Korea) (Figure S1). A threshold of −29–150 Hounsfield units (HU) was applied to identify muscle areas. The L3 skeletal muscle area represents whole-body muscle mass [19]. The SMD represents the muscle quality, which was assessed as the mean radiodensity (HU) of the entire cross-sectional muscle area at the L3 level, whereas the SMI represents the muscle quantity. The skeletal muscle area was divided by the square of the patient’s height to normalize the L3 SMI and was calculated as follows: lumbar skeletal muscle cross-sectional area (cm^2^)/height squared (m^2^). The detailed abdominal CT protocol is described in Figure S1.

### 2.3. Statistical Analysis

In this study, since no clear standard cutoff has been validated for muscle-related indicators, including the SMI and SMD, in cancer patients, we divided these variables into tertiles for the categorical analysis. Specifically, in the neoadjuvant chemoimmunotherapy (NACIT) group, the lowest tertile of SMD, which corresponded to values below 48 HU, was classified as indicative of a low SMD. In addition, in the neoadjuvant chemotherapy-only (NACT) group, the lowest tertile of SMD, which corresponded to 44 HU, was applied as the cutoff for the analysis.

The chi-squared and Fisher’s tests for categorical variables were used to compare the clinicopathological differences between the groups. The correlation analysis between the clinical variables was performed using a Pearson or Spearman’s correlation. The Jonckheere–Terpstra test was applied to identify an ordered trend of clinical variables between the groups. Univariate analyses for survival were performed using the Kaplan–Meier method and the log-rank test or log-rank test for trend. Multivariable logistic regression analyses were performed to identify the independent predictors of a pCR. Cox proportional hazards regression analyses were used for multivariate models to verify the predictive or prognostic values of the clinical variables. Statistical significance was set at *p* < 0.05. All statistical analyses were performed using R version 4.4.2 (R Foundation for Statistical Computing, Vienna, Austria; http://www.r-project.org).

The time from the start of the treatment to death from any cause or the last follow-up date was defined as the overall survival. The definition of EFS was adapted from the Standardized Definitions for Efficacy End Points in Neoadjuvant Breast cancer Clinical Trials (NeoSTEEP) [34]. Event-free survival (EFS) was defined as the time from the start of the neoadjuvant treatment to the first occurrence of any of the following events: disease progression precluding definitive surgery, local or distant recurrence, development of a second primary cancer, or death from any cause.

### 2.4. PD-L1 Expression and Combined Positive Score

PD-L1 immunohistochemistry assays were performed using 22C3 pharmDx (Dako, Santa Clara, CA, USA) on the Dako Autostainer Link 48 with an EnVision DAB Detection System (Agilent Technologies, Santa Clara, CA, USA), according to the manufacturer’s instructions.

The CPS was calculated by dividing the total number of PD-L1-positive cells (tumor cells, lymphocytes, and macrophages) by the total number of tumor cells and multiplying the result by 100.

## 3. Results

### 3.1. Baseline Characteristics

Between March 2021 and March 2024, a total of 144 patients who met the inclusion and exclusion criteria were recruited. Among them, 102 patients received NACIT, while 42 patients received NACT.

All patients received a uniform chemotherapy backbone that consisted of four cycles of weekly paclitaxel (80 mg/m^2^) combined with three-weekly carboplatin (AUC 5), followed by four cycles of three-weekly doxorubicin (60 mg/m^2^) and cyclophosphamide (600 mg/m^2^); in the NACIT group, three-weekly pembrolizumab was additionally administered for up to eight cycles. The median duration of the follow-up was 15.2 months (95% CI, 13.6 to 18.9 months). Table S1 provides a summary of the baseline characteristics of the patients.

### 3.2. Association Between Muscle-Related Indices and Clinical Factors

**Figure 1:**
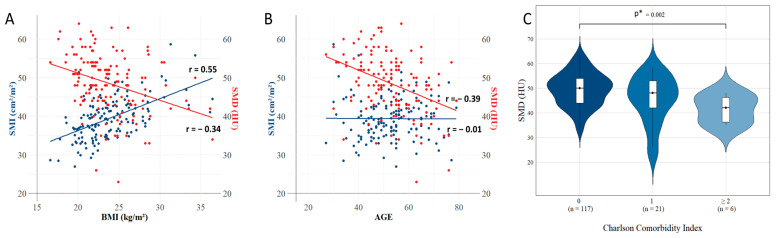
Correlation analysis between muscle-related indices and clinical factors. (**A**) The BMI showed a significant positive correlation with the SMI (r = 0.55, *p* < 0.001) and a significant negative correlation with the SMD (r = −0.34, *p* < 0.001). (**B**) Age was negatively correlated with the SMD (r = −0.39, *p* < 0.001) but showed no significant correlation with the SMI (r = −0.01, *p* = 0.945). (**C**) When the patients were categorized into three groups based on the Charlson Comorbidity Index (CCI) (0, 1, and ≥2), the SMD exhibited a significant decreasing trend with increasing CCI scores (Jonckheere–Terpstra test, *p* = 0.002). SMI, skeletal muscle index; BMI, body mass index; SMD, skeletal muscle density; HU, Hounsfield unit; CCI, Charlson Comorbidity Index. * indicates *p* < 0.05.

A total of 144 patients were included in the analysis, and the relationships between muscle-related indices and clinical factors, including the BMI, age, comorbidity, and ECOG performance status (PS), were assessed (Figure 1).

The SMI demonstrated a significant positive correlation with the BMI (r = 0.55, *p* < 0.001), whereas the SMD exhibited a significant negative correlation with the BMI (r = −0.34, *p* < 0.001). When the patients were stratified by BMI < 25 vs. BMI ≥ 25, the SMD was significantly higher in the BMI < 25 group compared with the BMI ≥ 25 group (49.71 vs. 45.83 kg/m^2^, *p* = 0.005). However, the SMI and SMD were not significantly correlated (r = −0.08, *p* = 0.369).

Age was significantly negatively correlated with the SMD (r = −0.39, *p* < 0.001) but showed no significant association with the SMI (r = −0.01, *p* = 0.945). These findings suggest that the muscle quality represented by the SMD declined with age, whereas the muscle quantity represented by the SMI remained relatively unaffected. When classified by the Charlson Comorbidity Index (CCI) scores (0, 1, and ≥2), the SMD exhibited a significant decreasing trend with an increasing comorbidity burden (Jonckheere–Terpstra test, *p* = 0.002) [35].

When patients were grouped by the ECOG PS (0 vs. ≥1), the SMD was numerically higher in the ECOG PS 0 group compared with those with ECOG PS ≥ 1 (49 vs. 48 HU, *p* = 0.189), which showed no significant difference. However, although the number was small, a subset of patients in the ECOG PS > 1 group exhibited notably lower SMD values (Figure S2). These results suggest that in the early-stage TNBC patients that underwent NAC, a younger age, lower BMI, and fewer comorbidities were associated with higher SMD levels.

### 3.3. Baseline Characteristics of Chemoimmunotherapy Group

Among the 102 patients in the NACIT group, the patients were divided into three groups according to the SMD tertiles. The tertiles were defined with cutoff values of 48 and 53 HU, which classified the SMD as low (<48 HU), medium (48 ≤ SMD < 53 HU), and high (≥53 HU). The pretreatment clinical variables were compared across the three groups. The proportion of premenopausal patients was higher in the high- and medium-SMD groups compared with the low-SMD group, while no other significant differences in the pretreatment variables were observed between the three groups (Table S2).

Subsequently, patients were stratified into two groups based on the lowest tertile SMD threshold of 48 HU, which resulted in a high-SMD group (n = 68) and a low-SMD group (n = 34). The baseline characteristics of the NACIT group are summarized in Table 1. The high-SMD group had a significantly higher proportion of patients under 65 years of age and a higher prevalence of premenopausal patients compared with the low-SMD group. The germline BRCA status was assessed in 83 out of 102 patients (81.4%), and pathogenic or likely pathogenic variants were more prevalent in the low-SMD group than in the high-SMD group but were not statistically significant (*p* = 0.062). The relative dose intensity (RDI) was significantly higher in the high-SMD group compared with the low-SMD group (89.4% vs. 82.5%, *p* = 0.003). Additionally, the BMI was lower in the high-SMD group but did not reach statistical significance (23.26 vs. 24.62, *p* = 0.068). The SMI did not differ significantly between the two groups.

The proportion of patients that achieved a pCR was numerically higher in the high-SMD group (63.2% vs. 44.1%, *p* = 0.066), although this difference did not reach statistical significance. Moreover, while no significant differences were observed between the groups regarding the rates of breast-conserving surgery and mastectomy, patients in the high-SMD group were significantly less likely to undergo axillary lymph node dissection compared with those in the low-SMD group (5.9% vs. 18.8%, *p* = 0.045). This finding may reflect the higher pCR rate observed in the high-SMD group.

Patients in the NACT group were significantly older and had a higher proportion of post-menopausal patients compared with the NACIT group. However, there was no significant difference in the RDIs between the two cohorts. The clinical characteristics of the NACIT group are provided in Table S1. Among the 42 patients in the NACT group, 16 (38.1%) achieved a pCR, which was lower than the pCR rate in the NACIT group (56.9%).

### 3.4. Adverse Event and RDI

In the NACIT group, 17 patients (16.7%) experienced treatment discontinuation of at least one agent during the neoadjuvant phase. Among them, 2 patients discontinued treatment due to local disease progression, while the remaining 15 patients (14.7%) discontinued due to adverse events.

Of these, 11 patients (10.8%) discontinued treatment due to immune-related toxicity, including pneumonitis (n = 3), adrenal insufficiency (n = 4), nephritis (n = 1), severe hyperthyroidism (n = 1), severe cutaneous adverse reaction (SCAR) (n = 1), and a sarcoid-like reaction (n = 1).

Among these 15 patients, 4 were able to complete the neoadjuvant treatment by continuing cytotoxic agents without pembrolizumab, whereas the remaining 11 patients discontinued all the neoadjuvant treatment (Table S3). Notably, all four patients who were able to complete the treatment belonged to the high-SMD group. Among the 11 patients who completely discontinued the neoadjuvant treatment due to toxicity, 3 belonged to the high-SMD group, while 8 were in the low-SMD group, which demonstrated a significantly higher rate of total treatment discontinuation in the low-SMD group (4.4% vs. 23.5%, χ^2^ *p* = 0.003).

Furthermore, in the NACIT group, we analyzed the correlations between the muscle-related indicators and the RDI. The SMD exhibited a significant positive correlation with the RDI (Spearman’s ρ = 0.31, *p* = 0.002), whereas the SMI showed no significant correlation. Additionally, age (ρ = −0.42, *p* < 0.001), the ECOG PS (ρ = −0.34, *p* < 0.001), and the CCI (ρ = −0.25, *p* = 0.010) demonstrated significant negative correlations with the RDI.

### 3.5. Univariate and Multivariable Regression Analysis for pCR

A regression analysis was performed in the NACIT group (n = 102) to identify the predictors of a pCR (Table 2). In the univariate analysis, the combined positive score (CPS) and SMD as continuous variables were significantly associated with a pCR, while the histologic grade and Ki-67 showed a *p*-value < 0.1. However, the RDI did not demonstrate a significant association with a pCR.

In the multivariable analysis, only the CPS and SMD remained as independent predictors of a pCR. For every 10-point increase in the CPS, the odds ratio (OR) for achieving a pCR was 1.38 (95% CI: 1.07–1.85, *p* = 0.019). Additionally, for every 1-unit increase in the SMD, the OR for a pCR was 1.11 (95% CI: 1.04–1.19, *p* = 0.003). The effect of the SMD on a pCR was further analyzed at different increments, showing that a 5-unit increase in the SMD corresponded to an OR of 1.67 (95% CI: 1.20–2.40, *p* = 0.003), while a 10-unit increase in the SMD resulted in an OR of 2.78 (95% CI: 1.45–5.74, *p* = 0.003).

Since the CPS and SMD were identified as significant predictors of a pCR in the NACIT group, these factors were evaluated in the NACT group. In the univariate regression analysis for a pCR conducted in 42 patients, the SMD did not show statistical significance. Among the 31 patients with available CPS data, the univariate regression analysis revealed that the CPS was not significantly associated with a pCR, although a marginal *p*-value was observed (*p* = 0.067).

### 3.6. Differences in pCR Based on CPS and SMD Groups

**Figure 2:**
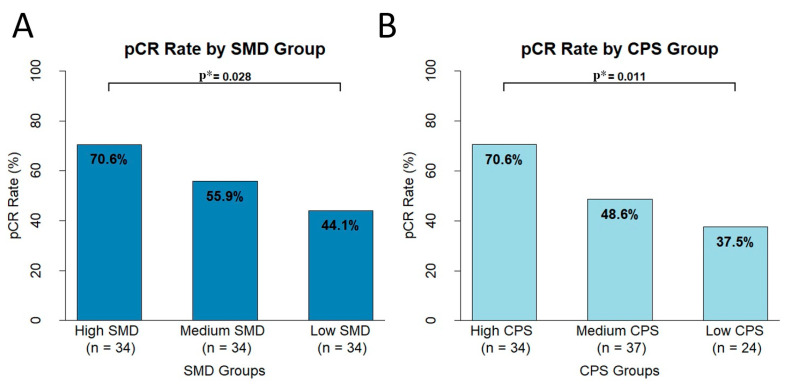
Pathologic complete response (pCR) rates in patients who underwent neoadjuvant chemoimmunotherapy (n = 102), stratified by skeletal muscle density (SMD) tertiles. The SMD tertiles were defined with cutoff values at 48 and 53, while the CPS tertiles were set at 10 and 20. The CPS-based analysis included 95 patients due to indeterminate CPSs in 7 cases. (**A**,**B**) pCR rates in the overall cohort showed a significant association with a higher SMD and CPS. Statistical significance was assessed using the chi-square test for trend. * indicates *p* < 0.05.

Since the SMD and CPS were identified as independent predictors of a pCR, both continuous variables were categorized into three tertile-based groups (low, medium, and high) to evaluate the differences in the pCR rates across the categories (Figure 2). The characteristics of the SMD tertiles were defined with cutoff values at 48 and 53, while the CPS tertiles were set at 10 and 20. Consequently, the SMD was classified as low (<48), medium (48 ≤ SMD < 53), and high (≥53), and the CPS as low (0 ≤ CPS < 10), medium (10 ≤ CPS < 20), and high (≥20).

The analysis demonstrated that the pCR rates were the highest in the high-SMD group, followed by the medium- and low-SMD groups, indicating a significant increasing trend between the SMD and pCR rates (χ^2^ for trend, *p* = 0.028). Similarly, the CPS-based stratification showed a significant trend, with the highest pCR rates observed in the high-CPS group, followed by the medium- and low-CPS groups (χ^2^ for trend, *p* = 0.011). Notably, among the 12 patients who met both the high-SMD and high-CPS criteria, 11 (91.7%) achieved a pCR.

### 3.7. Univariate and Multivariate Analysis for Event-Free Survival

**Figure 3:**
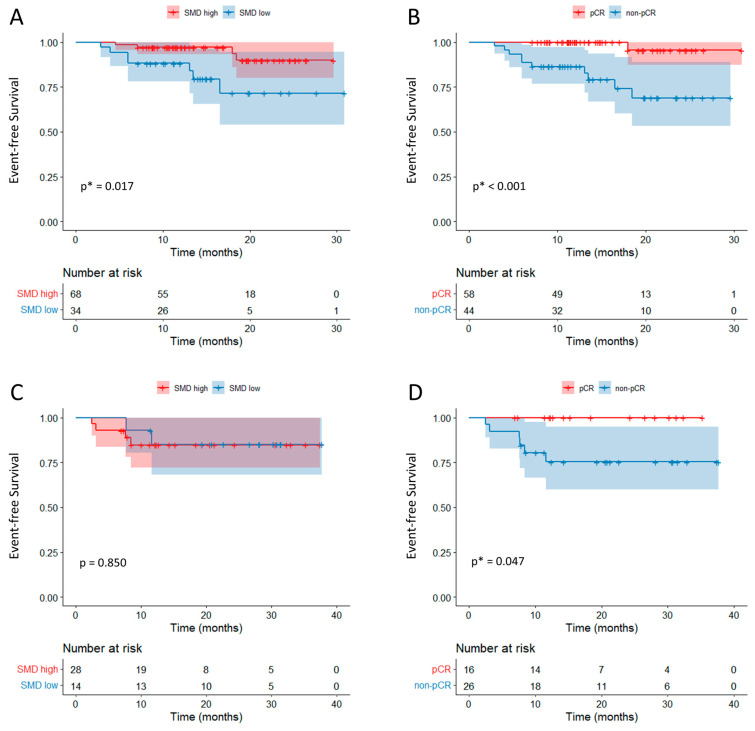
Kaplan–Meier analysis of the event-free survival (EFS) in patients who received neoadjuvant chemoimmunotherapy (NACIT) and chemotherapy (NACT). (**A**,**B**) In the NACIT group, patients in the high-SMD group exhibited a significantly longer EFS compared with those in the low-SMD group (*p* = 0.017). Additionally, the EFS was significantly longer in the patients who achieved a pathologic complete response (pCR) compared with those who did not (non-pCR) (*p* < 0.001). (**C**,**D**) In the NACT group, the EFS was significantly longer in patients who achieved a pCR (*p* = 0.047), whereas no significant difference in the EFS was observed between the high-SMD and low-SMD groups. * indicates *p* < 0.05.

The EFS was analyzed in the NACIT group (n = 102) using the Kaplan–Meier method, with patients stratified based on the lowest tertile of SMD. The high-SMD group exhibited a significantly longer EFS compared with the low-SMD group (*p* = 0.017, median EFS—not reached) (Figure 3). Furthermore, additional analyses of the progression-free survival (PFS) and overall survival (OS) revealed that the high-SMD group had a significantly longer PFS and OS than the low-SMD group (Figure S3).

Additionally, the EFS was compared between patients who achieved a pCR and those who did not, demonstrating a significantly longer EFS in the pCR group (*p* < 0.001).

Further univariate and multivariate analyses were performed to evaluate the prognostic factors for the EFS. In the univariate analysis, the SMD and pCR showed statistical significance, while age and the RDI demonstrated a *p*-value < 0.1. However, in the multivariate analysis, only a pCR remained an independent prognostic factor for the EFS (Table 3).

In the NACT group, the SMD groups classified by the lowest tertile did not show a significant difference in the EFS between the SMD high and low groups (*p* = 0.850) (Figure 3C). However, similar to the NACIT group, the patients in the pCR group demonstrated a significantly better EFS compared with those in the non-pCR group (*p* = 0.047) (Figure 3D).

## 4. Discussion

In this study, we demonstrated that the SMD, measured via CT, was associated with indicators of physical reserve, such as age and comorbidities, in stages II-III TNBC patients that underwent NAC consisting of paclitaxel/carboplatin followed by doxorubicin/cyclophosphamide with or without pembrolizumab. Additionally, among patients that received NACIT, both the SMD and CPS, analyzed as continuous variables, showed significant associations with a pCR.

Physical reserve is a crucial factor in pretreatment evaluation for chemotherapy candidates, with age and the ECOG PS being the most representative indicators. However, chronological age alone is a simple yet limited pretreatment factor, as it does not adequately capture individual variations in biological aging and functional reserve. Furthermore, in early-stage TNBC patients undergoing NAC, the ECOG PS is usually 0 or 1, limiting its effectiveness as a discriminator of physical reserve. Moreover, the ECOG PS is highly subjective and evaluator-dependent [36]. In contrast, the SMD is a continuous variable that can be objectively measured via CT and has been shown in several studies to correlate with physical reserve indicators, such as age and the ECOG PS [16,37]. Our study confirmed these associations and further established a relationship between the SMD and comorbidities through the CCI. Thus, the SMD appears to reflect physical reserve in patients with early TNBC receiving NAC.

In our study, although the rate of adverse events leading to discontinuation was similar to that in the KN-522 trial, chemotherapy dose reductions occurred more frequently (69.6% of patients). Despite these modifications, efficacy outcomes, including a pCR, were comparable with KN-522, aligning with recent real-world data [38]. Notably, patients with a high SMD improved with proper management and continued NAC, suggesting that the SMD may represent a biomarker for predicting the ability to tolerate chemotherapy-related adverse events and maintain treatment completion during the neoadjuvant phase.

In several previous studies on early breast cancer, a low SMD has been interpreted as a reflection of intramuscular adiposity, which negatively affects chemotherapy tolerance, thereby reducing the dose intensity of adjuvant chemotherapy and ultimately impacts the treatment efficacy [22,23]. Our study, consistent with previous research, found that the SMD showed a modest but significant correlation with the RDI in the NACIT group, suggesting that this relationship may partially explain the association between the SMD and the treatment response. However, age exhibited a stronger correlation with the RDI, and while the SMD was an independent predictor of a pCR, the RDI and age were not. This may be attributed to the fact that the RDI does not account for pembrolizumab administration, and the KN-522 regimen may represent overtreatment in certain patients with a lower disease burden, leading to an unclear relationship between the RDI and a pCR. Furthermore, the SMD’s predictive value for the treatment response suggests that its influence extends beyond facilitating chemotherapy tolerance and maintaining the RDI.

Excessive fat accumulation within skeletal muscle is referred to as myosteatosis. In this study, considering ethnic differences and the fact that our study population comprised patients with early-stage TNBC, we did not adopt the conventional myosteatosis cutoff values used in prior studies. Instead, we analyzed the SMD using tertiles and as a continuous variable. By employing a tertile-based approach, we minimized the bias that can arise from arbitrarily selected cutoff points. This approach further enabled us to demonstrate a clear dose–response relationship, thereby supporting the reliability of the SMD as a potential predictive marker for a pCR. Using the most commonly used criteria (SMD < 41 HU for BMI < 25 kg/m^2^ and SMD < 33 HU for BMI ≥ 25 kg/m^2^) [39], seven patients in the NACIT group and three in the NACT group met the definition, while only 14.3% of the NACIT group achieved a pCR and none in the NACT group. Myosteatosis is closely linked to chronic systemic inflammation and CD8^+^ T cell dysfunction within the TIME [31,40], which may reduce the clinical benefits of immunotherapy [40,41,42]. Additionally, considering that immunogenic cell death (ICD)—which stimulates adaptive immunity and tumor-specific T-cell activation [43]—is considered the primary mechanism underlying the efficacy of NACIT, myosteatosis might negatively impact these immune activation pathways. Conversely, an adequate muscle quality and sufficient physical reserve might represent essential host factors required for effective ICD and adaptive immune activation, potentially explaining why a higher muscle quality could translate into greater treatment efficacy.

In the NACT group, the association between the SMD and a pCR or prognostic value was not as clear compared with the NACIT group. Although there was no significant difference in the SMD between the two cohorts, the NACIT group showed a numerically higher value (49.32 HU vs. 46.76 HU, *p* =0.078). However, the RDI did not differ between the groups, which may be related to the known higher toxicity of chemotherapy combined with a PD-1 inhibitor compared with chemotherapy alone [33]. This increased toxicity in the NACIT group may have strengthened the association between the SMD, treatment tolerability, and response. Additionally, the relatively small sample size of the NACT group may have limited the ability to detect a significant predictive value of the SMD. Therefore, further studies including a larger number of patients in the chemotherapy-only group are warranted.

In our study, the multivariate analysis demonstrated that achieving a pCR was the only independent predictor of improved survival outcomes, whereas the SMD was not independently significant. The powerful prognostic impact of a pCR on survival was reaffirmed in our study; the relatively short follow-up duration likely limited the identification of other significant prognostic factors. Additionally, in the NACIT group, patients underwent various subsequent treatments after NAC, such as adjuvant pembrolizumab, capecitabine, or participation in clinical trials, potentially influencing the survival outcomes, and thus, affecting the evaluation of the SMD as an independent prognostic factor. Nevertheless, given the demonstrated association between the SMD and a pCR, a longer follow-up period may reveal SMD as a significant predictor for EFS. Therefore, further investigation is necessary to clarify the prognostic significance of SMD in TNBC patients treated with NACIT. Moreover, evaluating dynamic changes in SMD throughout treatment and follow-up could provide additional prognostic insights.

PD-L1 expression, along with TILs, is a tumor microenvironment-related marker that is expressed as a continuous variable. PD-L1 expression correlates with stromal TIL levels, and high PD-L1 expression and stromal TIL levels have been associated with higher pCR rates [8,9]. However, both TILs and PD-L1 expression exhibit intratumoral and inter-observer heterogeneity, posing significant limitations [44,45]. In contrast, SMD is a host factor that can be objectively, consistently, and easily measured via CT. The fact that the SMD’s odds ratio and statistical significance for a pCR were similar to those of the CPS, along with a clear trend when categorized into tertiles, suggests that SMD may serve as a predictive marker for pCR from a host factor perspective.

To our knowledge, this study is the first to investigate the relationship between the CT-based SMD and the treatment response to NACIT in TNBC patients. Our findings suggest that SMD, as an objective and reproducible marker, may be useful as a predictive marker for pCR as a host factor alongside a tumor microenvironment-related marker. Further validation in larger patient cohorts is warranted.

This study had several limitations. It was a retrospective analysis conducted at a single institution with 144 patients, and the results have not been externally validated. Therefore, caution is needed in interpreting the findings. Additionally, there is no universally agreed-upon cutoff for SMD, so we applied both tertile-based and continuous-variable approaches to enhance the data reliability. However, the limited sample size remains a constraint. Furthermore, although SMD has been linked to physical exercise in prior studies, we did not assess the correlation between the SMD and physical exercise in our study population, which is another limitation. Although the pretreatment PD-L1 CPS was analyzed and its clinical significance explored, a comparative analysis with TILs could not be performed, which we also acknowledge as a limitation.

## 5. Conclusions

The CT-measured SMD reflected the physical reserve in TNBC patients that received NAC. Alongside the CPS, SMD may serve as a predictive marker for NACIT efficacy, warranting further validation in larger cohorts.

## Figures and Tables

**Table 1:** Baseline characteristics.

Variables		High SMD (≥48)		Low SMD (<48)		
		n = 68		n = 34		*p*-Value
Pretreatment						
Age (years)	53 (IQR 45~61)					
<65	88	62	91.2%	26	76.5%	0.042
≥65	14	6	8.8%	8	23.5%	
Menopausal state						
Pre-menopausal	55	43	63.2%	12	35.3%	0.008
Post-menopausal	47	25	36.8%	22	64.7%	
ECOG PS						
0	93	63	92.6%	30	88.2%	0.459
≥1	9	5	7.4%	4	11.8%	
CCI						
0	82	58	85.3%	24	70.6%	0.078
≥1	20	10	14.7%	10	29.4%	
Stage						
II	60	39	57.4%	21	61.8%	0.67
III	42	29	42.6%	13	38.2%	
Tumor size						
T1–2	78	54	79.4%	24	70.6%	0.322
T3–4	24	14	20.6%	10	29.4%	
Node metastasis						
Positive	68	47	69.1%	21	61.8%	0.458
Negative	34	21	30.9%	13	38.2%	
Differentiation						
Grades 1–2	14	10	14.7%	4	11.8%	0.684
Grade 3	88	58	85.3%	30	88.2%	
Ki-67	62 (IQR 46–77)					
<20	3	1	1.5%	2	5.9%	0.214
≥20	99	67	98.5%	32	94.1%	
Germline BRCA						
PV/LPV	9	4	5.9%	5	14.7%	0.062
Not detected/VUS	74	55	80.9%	19	55.9%	
Undetermined	19	9	13.2%	10	29.4%	
PD-L1 (CPS)	10 (IQR 10–25)					
<10	24	15	22.1%	9	26.5%	0.742
≥10	71	47	69.1%	24	70.6%	
Undetermined	7	6	8.8%	1	2.9%	
SMD (HU)	49.32 ± 7.18	53.38 ± 4.31		41.21 ± 4.27		<0.001
SMI (cm^2^/m^2^)	39.19 ± 5.31	39.04 ± 5.48		39.50 ± 5.00		0.674
BMI (kg/m^2^)	23.71 ± 3.46	23.26 ± 3.34		24.62 ± 3.56		0.068
Post-treatment						
Breast Surgery						
BCS	89	60	88.2%	29	90.6%	0.722
Mastectomy	11	8	11.8%	3	9.4%	
No surgery	2			2		
Axillary Surgery						
SLNB	90	64	94.1%	26	81.3%	0.045
ALND	10	4	5.9%	6	18.8%	
No surgery	2			2		
Pathologic complete response (pCR)						
pCR	58	43	63.2%	15	44.1%	0.066
Non-PCR	44	25	36.8%	19	55.9%	
RDI (%)	86.9 (IQR 80.0–98.1)	89.4 (IQR 82.4–100)		82.5 (IQR 70.0–90.6)		0.003

SMD, skeletal muscle density; IQR, interquartile range; ECOG PS, Eastern Cooperative Oncology Group Performance Status; CCI, Charson Comorbidity Index; PV, pathogenic variant; LPV, likely pathogenic variant; VUS, variant of unknown significance; PD-L1, programmed death-ligand 1; CPS, combined positive score; SMI, skeletal muscle index; BMI, body mass index; BCS, breast-conserving surgery; SLNB, sentinel lymph node biopsy; ALND, axillary lymph node dissection; RDI, relative dose intensity.

**Table 2:** Univariate and multivariable logistic regression analysis for pathologic complete response.

	Univariate			Multivariable		
Variables	OR	(95% CI)	*p*-Value	OR	(95% CI)	*p*-Value
Age	0.98	0.95–1.02	0.34			
Menopausal state (post- vs. pre-menopausal state)	1.55	0.71–3.44	0.275			
ECOG PS (0 vs. ≥1)	0.50	0.14–1.68	0.264			
CCI (0 vs. ≥1)	0.55	0.20–1.47	0.236			
Germline BRCA (ND/VUS vs. PV/LPV)	2.88	0.65–20.04	0.201			
Stage (II vs. III)	1.15	0.55–2.43	0.713			
T stage (T1,2 vs. T3,4)	0.67	0.41–1.06	0.09	0.46	0.15–1.32	0.154
Nodal status (negative vs. positive)	1.52	0.66–3.50	0.324			
Histologic grade (grades 1–2 vs. grade 3)	2.73	0.87–9.51	0.094	2.27	0.58–9.54	0.247
Ki-67 (per 10% increase)	1.20	0.98–1.46	0.076	1.01	0.99–1.04	0.214
CPS (per 10-point increase)	1.34	1.06–1.81	0.028	1.38	1.07–1.85	0.019
RDI (per 10% increase)	1.20	0.96–1.55	0.13			
BMI	0.91	0.80–1.02	0.112			
SMI	0.96	0.89–1.03	0.249			
SMD (per 10 HU increase)	2.56	1.42–4.95	0.003	2.78	1.45–5.74	0.003
SMD (per 5 HU increase)	1.59	1.19–2.23		1.67	1.20–2.40	
SMD (per 1 HU increase)	1.10	1.04–1.17		1.11	1.04–1.19	

Categorical variables are denoted as “former vs. latter”, with the former as the reference. Variables not otherwise specified were treated as continuous. ECOG PS, Eastern Cooperative Oncology Group Performance Status; CCI, Charson Comorbidity Index; ND, not detected; VUS, variant of unknown significance; PV, pathogenic variant; LPV, likely pathogenic variant; CPS, combined positive score; RDI, relative dose intensity; BMI, body mass index; SMI, skeletal muscle index; SMD, skeletal muscle density; HU, Hounsfield units.

**Table 3:** Univarate and multivariate analysis for event-free survival.

	Univariate			Multivariate		
Variables	HR	(95% CI)	*p*-Value	HR	(95% CI)	*p*-Value
Age (<65 vs. ≥65)	3.09	0.81–11.73	0.098	2.22	0.53–9.25	0.273
Menopausal state (post- vs. pre-menopausal)	0.43	0.13–1.47	0.177			
ECOG PS (0 vs. ≥1)	2.02	0.87–4.68	0.102			
CCI (0 vs. ≥1)	1.53	0.57–4.09	0.397			
Germline BRCA (ND/VUS vs. PV/LPV)	1.13	0.14–8.92	0.906			
Stage (II vs. III)	1.42	0.49–4.14	0.516			
T stage (T1,2 vs. T3,4)	1.41	0.76–2.61	0.275			
Nodal status (negative vs. positive)	1.41	0.37–5.33	0.611			
Histologic grade (grades 1–2 vs. grade 3)	0.77	0.17–3.56	0.736			
Ki-67 (per 10% increase)	1.00	0.75–1.34	0.993			
CPS (per 10-point increase)	0.71	0.42–1.23	0.222			
RDI (per 10% increase)	0.80	0.64–1.02	0.067	0.94	0.72–1.23	0.636
BMI	0.95	0.79–1.14	0.559			
SMI	1.01	0.91–1.13	0.811			
SMD (per 10 HU increase)	0.42	0.19–0.93	0.033	0.6	0.25–1.46	0.259
SMD (per 1 HU increase)	0.92	0.85–0.99		0.95	0.87–1.04	
pCR	0.07	0.01–0.55	0.012	0.1	0.01–0.85	0.035

Categorical variables are denoted as “former vs. latter”, with the former as the reference. Variables not otherwise specified are treated as continuous. ECOG PS, Eastern Cooperative Oncology Group Performance Status; CCI, Charson Comorbidity Index; ND, not detected; VUS, variant of unknown significance; PV, pathogenic variant; LPV, likely pathogenic variant; CPS, combined positive score; RDI, relative dose intensity; BMI, body mass index; SMI, skeletal muscle index; SMD, skeletal muscle density; HU, Hounsfield units.

## Data Availability

The data presented in this study are available in this article and Supplementary Materials.

## Supplementary Materials

The following supporting information can be downloaded from https://www.mdpi.com/article/10.3390/cancers17111768/s1, Figure S1: The schematic diagram of the deep learning-based muscle analysis method (ClariMetabo) and example of a muscle analysis report; Figure S2: Correlation analysis between skeletal muscle density and ECOG performance status; Figure S3: Progression-free survival and overall survival in the NACIT and NACT groups; Table S1: Baseline characteristics of the NACIT and NACT groups; Table S2: Baseline characteristics of the SMD tertile-based groups; Table S3: Toxicity leading to treatment discontinuation.

## Abbreviations

The following abbreviations are used in this manuscript:

TNBC	Triple-negative breast cancer
ER	Estrogen receptors
PR	Progesterone receptors
HER2	Human epidermal growth factor receptor 2
NAC	Neoadjuvant chemotherapy
pCR	Pathologic complete response
TIL	Tumor-infiltrating lymphocyte
PS	Performance status
SMI	Skeletal muscle index
SMD	Skeletal muscle density
KN-522	KEYNOTE-522
HU	Hounsfield units
NACIT	Neoadjuvant chemoimmunotherapy
NACT	Neoadjuvant chemotherapy only
EFS	Event-free survival
CCI	Charlson Comorbidity Index
RDI	Relative dose intensity
SLNB	Sentinel lymph node biopsy
SCAR	Severe cutaneous adverse reaction
CPS	Combined positive score
PFS	Progression-free survival
OS	Overall survival
ICD	Immunogenic cell death
