# Supplementary material for: Skeletal Muscle Density as a Predictive Marker for Pathologic Complete Response in Triple-Negative Breast Cancer Treated with Neoadjuvant Chemoimmunotherapy
Source: Cancers (Basel). 2025 May 25;17(11):1768. doi: 10.3390/cancers17111768 (PMC12153542; doi:10.3390/cancers17111768)
Supplement: Supplementary file 1 [file cancers-17-01768-s001.zip › Figure S1_SMD.pdf]

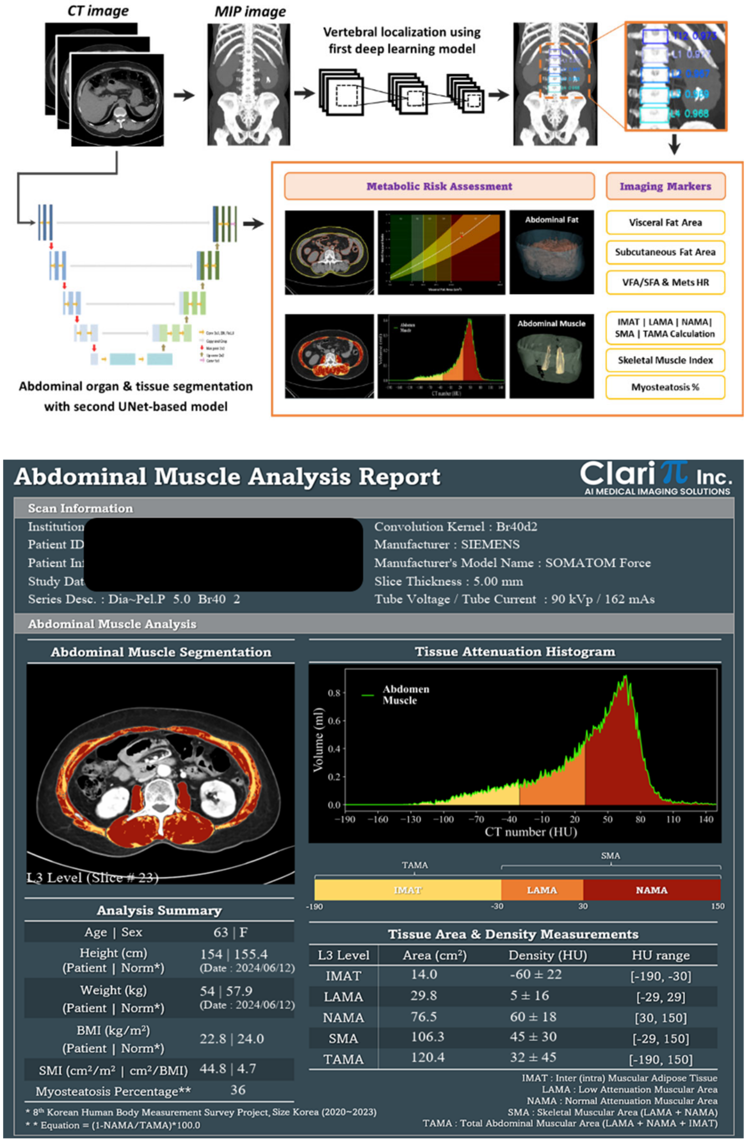

Figure S1. The schematic diagram of the deep learning-based muscle analysis method (ClariMetabo) and example of a muscle analysis report. ClariMetabo utilizes two deep learning (DL) models to quantify abdominal fat and muscle components in CT images. The first model, trained on MIP CT images from over 800 patient scans, localizes the vertebral body (T12–L4) with a categorical accuracy of 99.1%. The second model, a 2D U-Net trained on more than 40,000 CT images, automatically segments abdominal fat and muscle, achieving a Dice similarity coefficient of 0.96–0.98. To ensure consistency in image analysis, a predefined HU threshold (-29 to 150 HU) was applied, and an experienced radiologist visually reviewed the segmentation results to verify accuracy.

CT scans were acquired using multidetector CT scanners (SOMATOM Definition Edge and SOMATOM Definition Force, Siemens Healthineers, Erlangen, Germany; Discovery CT750 HD, GE Healthcare, Waukesha, WI, USA). Routine contrast-enhanced abdominopelvic CT protocol included a single portal venous phase. Patients received approximately 0.521 gI/kg of iodinated contrast medium of Ioversol, Iohexol, Iomeprol, or Iopromide at a rate of 2–2.5 mL/s followed by a 20 mL saline change via antecubital vein. Image acquisition began 75 s after the contrast medium injection. The scan range included the lung bases to 5 cm below the ischial tuberosities. Detailed scanning parameters were set as follows: kVp, 70–100 kVp; reference tube current-time product, 167–708 mAs; pitch, 0.7–1.1, rotation time, 0.5–0.6 s, and slice thickness, 5 mm without overlap. Automatic tube voltage selection and automatic tube current modulation were enabled in all scans.
