# Supplementary material for: Skeletal Muscle Density as a Predictive Marker for Pathologic Complete Response in Triple-Negative Breast Cancer Treated with Neoadjuvant Chemoimmunotherapy
Source: Cancers (Basel). 2025 May 25;17(11):1768. doi: 10.3390/cancers17111768 (PMC12153542; doi:10.3390/cancers17111768)
Supplement: Supplementary file 1 [file cancers-17-01768-s001.zip › Figure S2_SMD.pdf]

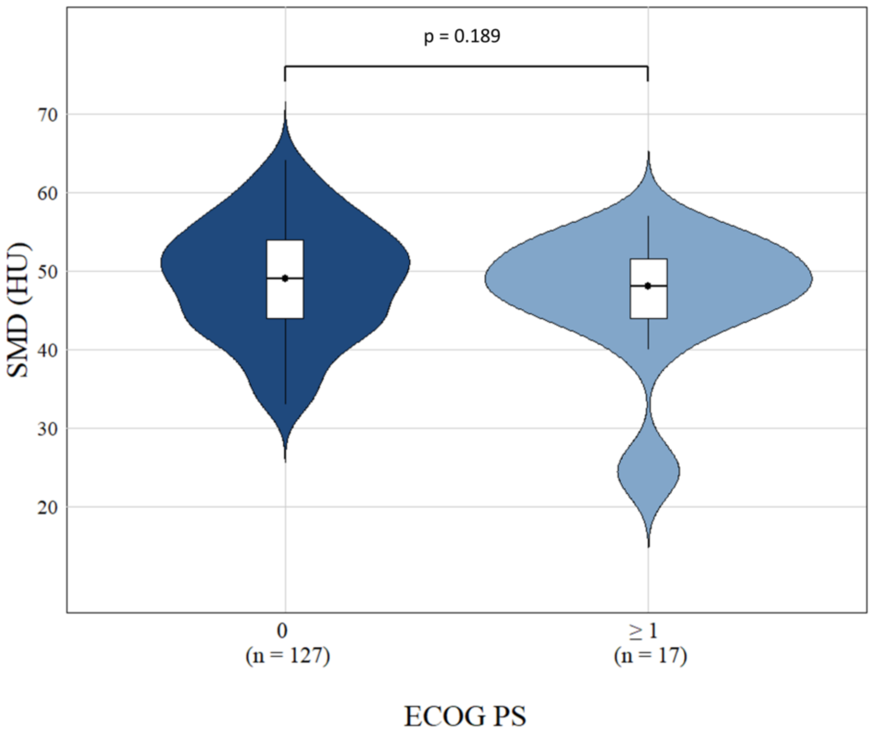

Figure S2. Correlation Analysis Between Skeletal Muscle Density and ECOG Performance Status.

SMD was numerically higher in the ECOG PS 0 group compared to those with ECOG PS ≥1 (49 vs. 48 HU, p = 0.189)
