# Supplementary material for: Skeletal Muscle Density as a Predictive Marker for Pathologic Complete Response in Triple-Negative Breast Cancer Treated with Neoadjuvant Chemoimmunotherapy
Source: Cancers (Basel). 2025 May 25;17(11):1768. doi: 10.3390/cancers17111768 (PMC12153542; doi:10.3390/cancers17111768)
Supplement: Supplementary file 1 [file cancers-17-01768-s001.zip › Figure S3_SMD.pdf]

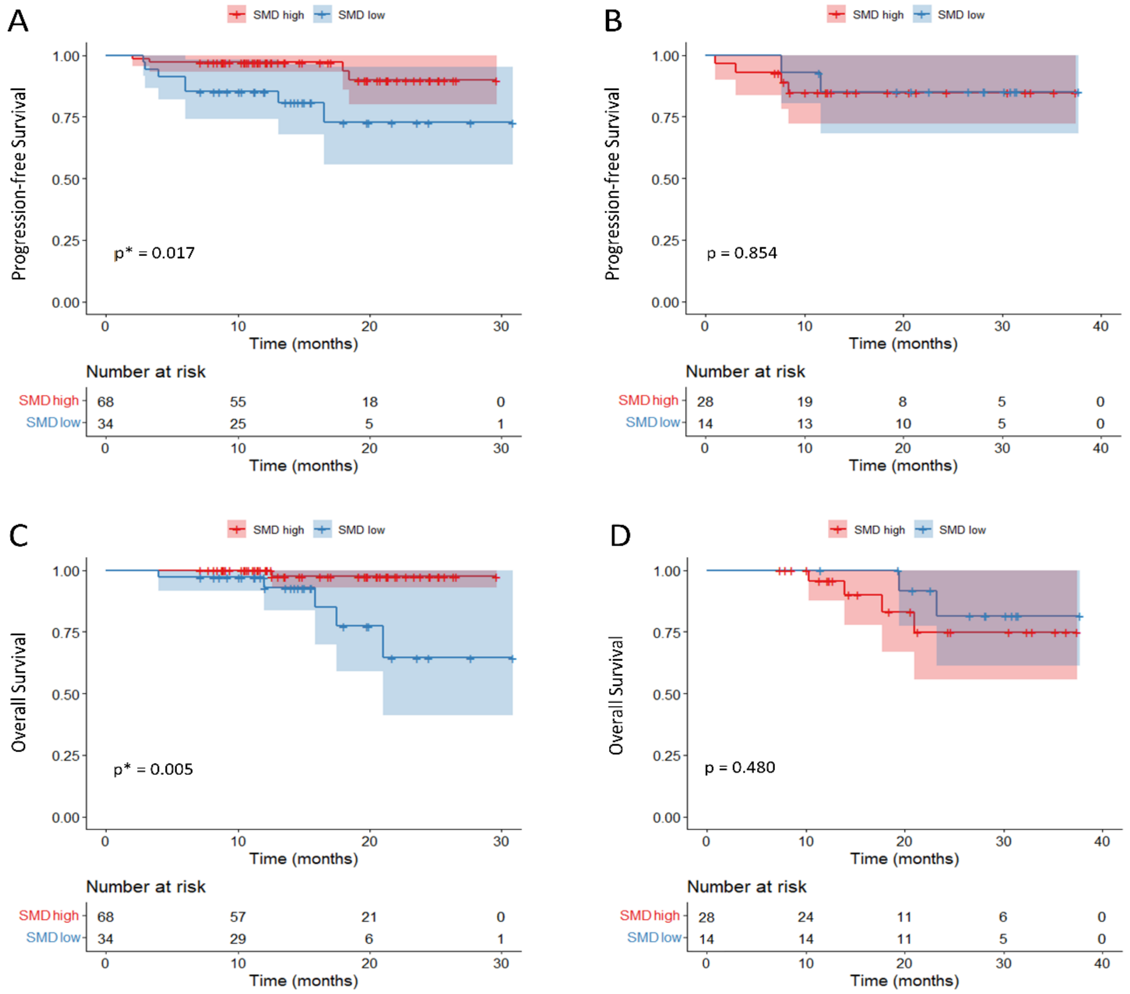

Figure S3. Progression-free survival and overall survival in the NACIT and NACT groups

(A, C) In the NACIT group, the high-SMD group showed significantly longer progression-free survival (PFS) and overall survival (OS) compared to the low-SMD group. (B, D) In the NACT group, there was no significant difference in PFS or OS between the high- and low-SMD groups.
