# Supplementary material for: Skeletal Muscle Density as a Predictive Marker for Pathologic Complete Response in Triple-Negative Breast Cancer Treated with Neoadjuvant Chemoimmunotherapy
Source: Cancers (Basel). 2025 May 25;17(11):1768. doi: 10.3390/cancers17111768 (PMC12153542; doi:10.3390/cancers17111768)
Supplement: Supplementary file 1 [file cancers-17-01768-s001.zip › Table S1_SMD proofreading.pdf]

Table S1. Baseline Characteristics of the NACIT and NACT Groups

**variables**	*number of patients*	*NACIT chemotherapy + pembrolizumab*		*NACT chemotherapy*		
	n= 144	n=102		n=42		p value
Age (years)	53 (IQR 45 ~ 60)					
< 65	121	88	86.3%	33	78.6%	0.251
≥ 65	23	14	13.7%	9	21.4%	
Menopausal state						
pre-menopausal	71	55	53.9%	16	38.1%	0.084
post-menopausal	73	47	46.1%	26	61.9%	
ECOG PS						
0	127	93	91.2%	34	81.0%	0.084
≥ 1	17	9	8.8%	8	19.0%	
C.C.I						
0	115	82	80.4%	35	83.3%	0.681
≥ 1	27	20	19.6%	7	16.7%	
Stage						
II	83	62	60.8%	21	50.0%	0.234
III	61	40	39.2%	21	50.0%	
Differentiation						
Grade 1-2	27	14	13.7%	13	31.0%	0.016
Grade 3	117	88	86.3%	29	69.0%	
Ki-67	61 (IQR 46 ~ 77)					
< 20	7	3	2.9%	4	9.5%	0.095
≥ 20	137	99	97.1%	38	90.5%	
PD-L1 (CPS)	10 (Range 0 - 100)*					
< 10	37	24	23.5%	13	31.0%	<0.001
≥ 10	89	71	69.6%	18	42.9%	
undetermined	18	7	6.9%	11	26.2%	
Breast Surgery						
BCS	127	89	89.0%	38	90.5%	0.794
Mastectomy	15	11	11.0%	4	9.5%	
no surgery	2					
Axillary Surgery						
SLNB	124	90	90.0%	34	81.0%	0.139

ALND	18	10	10.0%	8	19.0%	
no surgery	2					
Pathologic Complete Response (PCR)						
PCR	74	58	56.9%	16	38.1%	0.041
non-PCR	70	44	43.1%	26	61.9%	
Pembrolizumab cycle(s) administered		8 (Range 1 - 8)				
RDI	0.88 (IQR 0.80 - 1.00)	0.91 (IQR 0.80 - 0.99)		0.91 (IQR 0.82 - 1.00)		0.765
SMD (HU)	48.58 ±7.51	49.32 ± 7.18		46.76 ± 8.07		0.078
SMI (cm2/m2)	39.40 ± 5.41	39.19 ±-5.31		39.91 ± 5.59		0.478
BMI (kg/m2)	23.74 ±-3.57	23.71 ± 3.46		23.81 ± 3.89		0.888

SMD, skeletal muscle density; IQR, interquartile range; ECOG PS, Eastern Cooperative Oncology Group Performance Status; CCI, Charson Comorbidity Index; PD-L1, programmed death-ligand 1; CPS, combined positive score; BCS, breast-conserving surgery; SLNB, sentinel lymph node biopsy; ALND, axillary lymph node dissection; RDI, relative dose intensity; SMI, skeletal muscle index; BMI, body mass index
