# Supplementary material for: Skeletal Muscle Density as a Predictive Marker for Pathologic Complete Response in Triple-Negative Breast Cancer Treated with Neoadjuvant Chemoimmunotherapy
Source: Cancers (Basel). 2025 May 25;17(11):1768. doi: 10.3390/cancers17111768 (PMC12153542; doi:10.3390/cancers17111768)
Supplement: Supplementary file 1 [file cancers-17-01768-s001.zip › Table S2_SMD proofreading.pdf]

Table S2. Baseline characteristics of SMD tertile-based groups.

variables		*High SMD(SMD ≥ 54)*		*Medium SMD (48 ≤ SMD < 54)*		*Low SMD (< 48)*		
		n= 34		n=34		n=34		p value
Pretreatment								
Age (years)	53 (IQR 45 ~61)							
< 65	88	32	94.1%	30	88.2%	26	76.5%	0.099
≥ 65	14	2	5.9%	4	11.8%	8	23.5%	
Menopausal state								
pre-menopausal	55	22	64.7%	21	61.8%	12	35.3%	0.028
post-menopausal	47	12	35.3%	13	38.2%	22	64.7%	
ECOG PS								
0	93	32	94.1%	31	91.2%	30	88.2%	0.694
≥ 1	9	2	5.9%	3	8.8%	4	11.8%	
CCI								
0	82	30	88.2%	28	82.4%	24	70.6%	0.175
≥ 1	20	4	11.8%	6	17.6%	10	29.4%	
Stage								
II	60	19	55.9%	20	58.8%	21	61.8%	0.886
III	42	15	44.1%	14	41.2%	13	38.2%	
Differentiation								
Grade 1-2	14	5	14.7%	5	14.7%	4	11.8%	0.921
Grade 3	88	29	85.3%	29	85.3%	30	88.2%	
Ki-67	62 (IQR 46 - 77)							
< 20	3	0	0.0%	1	2.9%	2	5.9%	0.357
≥ 20	99	34	100.0%	33	97.1%	32	94.1%	
Germline BRCA								
PV/LPV	9	2	5.9%	2	5.9%	5	14.7%	0.174
Not detected / VUS	74	29	85.3%	26	76.5%	19	55.9%	
undetermined	19	3	8.8%	6	17.6%	10	29.4%	
PD-L1 (CPS)	10 (IQR 10 - 25)							
< 10	24	7	20.6%	8	23.5%	9	26.5%	0.908
≥ 10	71	24	70.6%	23	67.6%	24	70.6%	
undetermined	7	3	8.8%	3	8.8%	1	2.9%	
SMD (HU)	49.32 ± 7.18	56.79 ± 3.32		49.97 ± 1.66		41.21 ± 4.27		< 0.001
SMI (cm^2/m^2)	39.19 ± 5.31	39.03 ± 5.56		39.04 ± 5.49		39.50 ± 5.00		0.920

BMI (kg/m^2)	23.71 ± 3.46	22.94 ± 3.35		23.58 ± 3.35		24.62 ± 3.56		0.129
Post-treatment								
Breast Surgery								
BCS	89	30	88.2%	30	88.2%	29	90.6%	0.939
Mastectomy	11	4	11.8%	4	11.8%	3	9.4%	
no surgery	2					2		
Axillary Surgery								
SLNB	90	33	97.1%	31	91.2%	26	81.3%	0.097
ALND	10	1	2.9%	3	8.8%	6	18.8%	
no surgery	2					2		
Pathologic Complete Response (pCR)								
pCR	58	24	70.6%	19	55.9%	15	44.1%	0.087
non-PCR	44	10	29.4%	15	44.1%	19	55.9%	
RDI (%)	86.9 (IQR 80.0 - 98.1)	92.5 (84.2–100.0)		88.8 (78.2–98.9)		82.5 (70.9–90.2)		0.003

SMD, skeletal muscle density; IQR, interquartile range; ECOG PS, Eastern Cooperative Oncology Group Performance Status; CCI, Charson Comorbidity Index; PD-L1, programmed death-ligand 1; PV, pathogenic variant; LPV, likely pathogenic variant; VUS, variant of unknown significance, CPS, combined positive score; BCS, breast-conserving surgery; SLNB, sentinel lymph node biopsy; ALND, axillary lymph node dissection; RDI, relative dose intensity; SMI, skeletal muscle index; BMI, body mass index
