# Supplementary material for: Skeletal Muscle Density as a Predictive Marker for Pathologic Complete Response in Triple-Negative Breast Cancer Treated with Neoadjuvant Chemoimmunotherapy
Source: Cancers (Basel). 2025 May 25;17(11):1768. doi: 10.3390/cancers17111768 (PMC12153542; doi:10.3390/cancers17111768)
Supplement: Supplementary file 1 [file cancers-17-01768-s001.zip › Table S3_SMD.pdf]

Table S3. Toxicity leading to treatment discontinuation

Toxicity	Any component discontinuation	Pembrolizumab discontinuation only	Complete NAC discontinuation
	Total (High-SMD / low-)	Total (High-SMD / low-)	Total (High-SMD / low-)
**Immune-related Adverse Event (irAE)**			
Nephritis (acute kidney)	1 (0/G4)*		1 (0/G4)
Adrenal insufficiency	4 (G2G2/G2G2)	1 (G2/0)	3 (G2/G2G2)
Pneumonitis	3 (G2G3/G4)		3 (G2G3/G4)
Hyperthyroidism	1 (G3/0)	1 (G3/0)	
Sarcoidosis	1 (G2/0)	1 (G2/0)	
Severe cutaneous adverse reaction	1 (G3/0)	1 (G3/0)	
**Non- irAE**			
Peripheral neuropathy	1 (0/G3)		1 (0/G3)
Asthenia	3 (0/G2G3G3)		3 (0/G2G3G3)
**Total**	**15 (14.7%)**	**4 (3.9%)**	**11 (10.8%)**

*; The values in parentheses represent the distribution of cases between the High-SMD and Low-SMD groups, respectively. ‘Gx’ represents one patient at the corresponding grade.
